# Deep reinforcement learning for decision making of autonomous vehicle in non-lane-based traffic environments

**DOI:** 10.1371/journal.pone.0320578

**Published:** 2025-04-16

**Authors:** Yi Fei, Lu Xing, Lan Yao, Zhizhi Yang, Yujie Zhang

**Affiliations:** 1 Hunan Key Laboratory of Smart Roadway and Cooperative Vehicle-infrastructure Systems, Changsha University of Science and Technology, Changsha, China; 2 School of Traffic and Transportation Engineering, Changsha University of Science and Technology, Changsha, China; Van Lang University: Truong Dai hoc Van Lang, VIET NAM

## Abstract

Existing research on decision-making of autonomous vehicles (AVs) has mainly focused on normal road sections, with limited exploration of decision-making in complex traffic environments without lane markings. Taking toll plaza diverging area as an example, this study proposes a lateral motion strategy for AVs based on deep reinforcement learning (DRL) algorithms. First, a microscopic simulation platform is developed to simulate the realistic diverging trajectories of human-driven vehicles (HVs), providing AVs with a high-fidelity training environment. Next, a DRL-based self-efficient lateral motion strategy for AVs is proposed, with state and reward functions tailored to the environmental features of the diverging area. Simulation results indicate that the strategy can significantly reduce the diverging time of single vehicles. In addition, considering the long-term coexistence of AVs and HVs, the study further explores how the varying penetration of AVs with self-efficient strategy impacts traffic flow in the diverging area. Findings reveal that a moderate increase in AV penetration can improve overall traffic efficiency and safety. But an excessive penetration of AVs with self-efficient strategy leads to intense competition for limited road resources, further deteriorating operational conditions in the diverging area.

## Introduction

Recent investigations have indicated that most vehicle accidents are caused by human errors [1,2]. The decision-making abilities of human drivers are unstable due to the influence of environmental factors or their own driving characteristics. Autonomous vehicles (AVs), with higher perceptual capabilities and shorter response time, are widely recognized for having potential to improve both traffic efficiency and safety [3,4]. Decision-making is one of the most important abilities of AVs. It is expected that the maturation of autonomous driving technologies will enable vehicles to possess the capability to make correct driving decisions, providing people safe and efficient rides [5,6].

Vehicles often use lateral motions to obtain better traffic conditions [7]. Compared to longitudinal motion, lateral motion requires vehicles to consider more surrounding vehicles, making the decision-making process more complex [8,9]. Existing research on decision-making for the lateral motion of AVs mainly focuses on lane-changing maneuvers on regular road sections with clear lane markings. In such sections, AVs can divide their surroundings by lane markings and only need to obtain information about the vehicles ahead and behind their current and adjacent lanes. However, in non-lane-based complex traffic areas, such as intersections, diverging and merging areas, decision-making becomes more complex due to additional factors such as irregular road geometries, traffic signals, and varying queue lengths. Vehicles in these areas have longitudinal destinations but lack lateral constraints from lane markings, leading to conflicts from any angle [10,11]. How to ensure AVs make safe and effective lateral motion decisions in non-lane-based traffic areas is a significant challenge.

Two main issues need to be addressed. First, it is necessary to provide AVs with a high-fidelity training environment that can accurately reflect the real trajectories of surrounding human-driven vehicles (HVs) in non-lane-based areas. While microscopic traffic simulation techniques perform well on regular road sections, existing simulations for non-lane-based areas still adhere to one-dimensional, lane-based rules. These rules cannot capture the destination-driven, weak constraint motion features of HVs, failing to provide a realistic interaction for AVs. Second, depending on the environmental characteristics of non-lane-based areas, further research is needed to explore what information AVs should perceive and how to design their appropriate objectives to enable them to make safe and efficient lateral motion decisions.

Deep reinforcement learning (DRL) is a recently popular approach for modeling the lateral motion decisions of AVs, which combines the strengths of both deep learning (DL) and reinforcement learning (RL) methods [12,13]. DL can extract multiple key features from large volumes of driving behavior data and vehicle sensor data, capturing the complex relationships between these features to make human-like lateral motion decisions [14–16]. However, human driving decisions are based on limited observed information and include various individual driving styles, simulating human driving decisions may not always be a good choice for AVs. While RL can learn through interaction with a virtual simulation environment, utilizing trial and error and reward signals feedback, thus not relying on extensive labeled data [6,17]. Therefore, DRL-based models are suitable for training AVs to generate safer and more stable lateral motion decisions in complex non-lane-based traffic areas [18].

This paper selects the toll plaza diverging area, a typical representative of non-lane-based complex traffic areas, to explore how to make effective lateral motion decisions for AVs based on DRL-based models. In China, the types of toll plazas on highways are still dominated by traditional mainline toll plazas (TMTPs). The diverging area at TMTPs is a gradually widening structure without lane markings or physical dividers between the Electronic Toll Collection (ETC) and Manual Toll Collection (MTC) lanes. Vehicles with different toll collection types need to choose and drive to their matching toll lanes within a limited distance. And without lane markings, the motion of HVs in diverging areas follows a destination-driven and weak-constraint pattern. The combination of various complex factors has resulted in a high demand for lateral motions in the diverging area, further turning the area into a hotspot of vehicle conflicts [10,19]. Previous studies often assume that HVs follow lane-based rules in diverging areas, as modeled in commonly used traffic simulation software such as SUMO and VISSIM. This assumption fails to provide AVs with realistic interactions with HVs. Moreover, lateral motion of vehicles is simplified as an instantaneous process, leading to an underestimation of its impact on traffic flow in complex traffic areas.

Since AVs cannot be fully popularized in a short time, a long transitional period of a mixed traffic flow will exist [20]. Most studies on AV decision-making have only demonstrated the effectiveness of the proposed strategies in single AV scenarios, without considering their performance in mixed traffic flow with varying penetration rates. In China, an increasing number of vehicles are equipped with intelligent driving or advanced driver-assistance system. The lateral motion decisions based on these intelligent driving algorithms aimed at maximizing the benefits of single vehicles. When proposing a self-efficient strategy, it is essential to further investigate how the increasing number of AVs with self-efficient strategy might impact overall traffic flow, particularly in complex traffic environments. Therefore, this paper aims to propose a DRL-based method on for AVs to make safe and efficient lateral motion decisions at toll plaza diverging areas. The major methodological contributions of this study are: (1) Providing AVs with realistic driving scenarios featuring complex interactions by proposing a micro-simulation approach. It can accurately replicate the weak-constraint motions of HVs at toll plaza diverging areas, serving as a high-fidelity training environment for AV decision-making. (2) Designing DRL functions tailored to diverging areas, where the state function quantifies the surrounding environment without lane markings and extracts key information for perception and decision, while the reward function is designed to guide the AVs select toll lanes with shorter queue lengths while penalizing aggressive lateral motions. (3) Moreover, this study also contributes to investigating how the varying penetration rate of AVs with the proposed strategy influences traffic efficiency and safety in the diverging area.

## Literature review

The lateral motion decision-making models of vehicles can be broadly classified into rule-based models and learning-based models. Rule-based models simulate lateral motion decisions by establishing some explicit rules, assuming that vehicles will take lateral maneuvers once these conditions are satisfied, such as the Gipps model, the MOBIL model, and utility-based models [21,22]. These models can provide interpretability but are limited to a finite set of lateral motion scenarios, making them inadequate for training AVs to handle complex traffic situations [23]. Researchers are gradually shifting to learning-based decision-making models with better generalization performance. In recent years, many DRL-based lateral motion decision models have been proposed, incorporating various designs in terms of input information, reward functions, and model structures [ 3,5,24]. Improvements from the input information usually focus on selecting state variables or adding extra networks to enhance the capture of critical information. The design of reward functions aims to train the agent to combine multiple objectives, such as macroscopic and microscopic objectives, or efficiency, safety, and comfort. Model structure improvements mainly involve adding additional constraints to the model to ensure safer and more stable learning for the agent, or integrating other objectives, such as lateral motion trajectory planning, to ensure that the vehicle completes the lateral motion process in an expected manner.

Among various DRL-based methods, deep Q-network (DQN) is commonly used for lateral motion decisions of AVs as it is suitable for addressing discrete action spaces (e.g., left LC, right LC, keep current lane). For example, Li [3] proposed a DQN model integrated risk assessment to prompt the AVs to learn the LC decision strategy with the minimum risk. Their lane-changing strategies can prevent the AVs from collisions with crowded static obstacles dynamic vehicles. Wang [25] adopted a harmonious LC strategy for AVs based on the DQN. The model considers both the delay of an individual AV and the overall traffic efficiency on the road segment. Wang [26] designed a safe and efficient driving strategy for AVs that combined the lateral decision-making and rule-based trajectory modification. Nevertheless, DQN sometimes may overestimate the value of certain actions when computing target Q-values, leading to instability and convergence issues. Based on DQN, the Double Deep Q-Network (DDQN) and Dueling DQN are proposed to address these challenges. DDQN employs two networks to calculate target Q-values, one for action selection and another for target Q-value estimation, thereby reducing the overestimation problem. Dueling DQN enhances performance by decomposing the Q-value function into separate estimators for state value and action advantage, improving the learning of state-action values. For example, Cui [27] proposed a comprehensive decision-making model for both lateral and longitudinal motions of AVs in a multi-lane highway environment. Compared with rule-based model (MOBIL & IDM), the DRL-based models (DQN, DDQN, and Dueling DQN) can achieve better performance in driving safety and efficiency. Liu [28] introduced a DDQN-based method to enhance driving safety and fuel economy by controlling the lane changes and speed of AVs. Simulation results showed that the DRL-based driving strategy saved 24% more fuel compared to the benchmark tests.

DRL models can be applied not only to upper lane-changing decision-making but also to the lower-level control of the lane-changing process. For example, Wang et al. [29] developed a reinforcement learning framework integrated with collision avoidance strategies to coordinate longitudinal and lateral control of connected autonomous vehicles (CAVs), effectively mitigating congestion and improving traffic efficiency in mandatory lane-change scenarios. Guo et al. [17] proposed a multi-agent Transformer-based deep deterministic policy gradient (MA-TDDPG) method to model the coupled driving behavior of a lane-changing vehicle and its follower. Wang et al. [30] also introduced a deep reinforcement learning-based lane-changing model that trains AVs to complete lane changes in interactions with various human driving behaviors by controlling their steering angle and acceleration. Wang et al. [31] formulated a lane-changing strategy based on deep deterministic policy gradient (DDPG), enabling continuous action control in model-free dynamic driving environments. Their approach employs reward functions for AVs’ lateral control actions, positional deviations, and maneuvering time, guiding vehicles to perform safe and comfortable lane changes.

Overall, existing research on lateral motion decisions based on DRL methods mainly focuses on lane-based traffic scenarios, lacking the generalization for different road structures. And they often make idealized assumptions about the number, positions, and behaviors of surrounding vehicles, assuming these vehicles to be either stationary or moving at constant speeds. They fail to provide AVs with realistic driving scenarios dynamically interacting with HVs. The decision-making capability of AVs still needs further verification in non-lane-based complex traffic areas. Additionally, further investigation is needed to understand the impact of AVs’ lateral motion strategies on mixed traffic flow dynamics.

## Methodology

**Fig 1:**
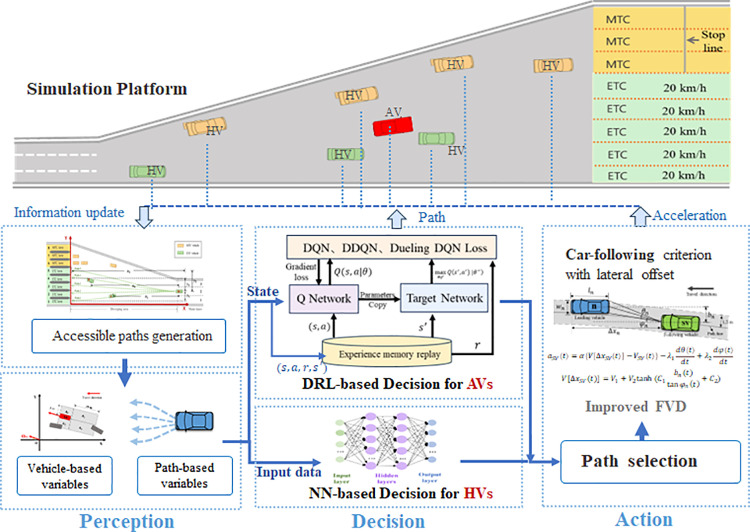
Illustration of framework.

This study proposes DRL-based methods for the lateral motion decision-making of AVs in non-lane-based traffic areas. Taking toll plaza diverging area as a case, the AVs are trained for selecting their target toll lanes during the diverging process in an efficient and safe way. The framework of this study is illustrated in Fig 1.

The upper part of the figure presents a diagram of our simulation platform for the TMTP diverging area. This platform incorporates a novel micro-simulation method to capture the weak-constraint motion characteristics of HVs in the diverging area, thereby providing AVs with a more realistic training environment. The lower half of Fig 1 depicts the driving behavior process in the diverging area. The diverging process for both HVs and AVs is divided into three components: perception, decision, and action. In the perception layer, the observations contain the vehicle-based information and path-based information of vehicle’s available target toll lanes. In the decision layer, AVs select their target toll lanes based on DRL methods, while HVs make decisions based on a calibrated neural network model. The performance of several classic DRL-based models for discrete decision-making are compared. In the action layer, an improved Full Velocity Difference (FVD) car-following model is used to describe the staggered car-following behavior during the diverging process.

The following will provide a detailed introduction to the principles of the DRL models and its related settings, and the micro-simulation method and simulation platform will be introduced in the next section.

### DRL model

The process of making lateral motion decisions based on DRL methods can be regarded as a Markov decision process (MDP). It is used to optimize sequential decision-making tasks by allowing an RL agent to interact with the environment [32]. An MDP can be characterized via a 4-tuple $\left(S,A,P,R\right)$, where *S* denotes the set of state, *A* is the set of the actions, *P* presents the transition probabilities, $R\left(S,A\right)$ represents the reward function. Specially, at each time step *t*, the agent observes the current state $s_{t}\in S$, and selects an action $a_{t}$ from a set of possible actions *A*based on a policy $\pi (a_{t}|s_{t})$, which maps state $s_{t}$ to corresponding action $a_{t}$. And at the same time, the methodology provides the agent with a reward $r_{t}$ and a transition to the subsequent state $s_{t+1}$. This iterative process continues until the agent reaches to the terminal state.

The goal of RL is to maximize the total reward $G_{t}$, which is defined as:


$$G_{t}=R_{t}+\gamma R_{t+1}+\gamma^{2}R_{t+2}+\ldots =\overset{\infty }{\underset{k=1}{\sum }}\gamma^{k}R_{t+k}$$ (1)

where *γ* is the discount factor, with 0≤γ≤1. The discount factor *γ* is important for shaping the influence of future rewards on an agent’s decision-making process. When γ=0, the agent prioritizes immediate rewards and doesn’t consider future consequences. And when γ=1, the agent considers the cumulative sum of all future rewar when evaluating each action.

The high-dimensional input and the delay between actions and rewards pose challenges to traditional RL-based methods. The integration of DL enables DRL to show great potential in addressing coupled nonlinear control and decision-making problems. In this study, the adopted DRL algorithms are DQN, DDQN, and Dueling DQN. Their performances on providing safe and efficient land change decision are compared further.

DQN is a variant of the Q-learning algorithm. Q-learning is a value-based RL algorithm used to approximate the optimal action-value function (Q-function) for an agent in a Markov Decision Process (MDP). The state-action value function $Q^{\pi }\left(s,a\right)$ for policy *π* is defined as:


$$Q^{\pi }\left(s,a\right)=E_{\pi }\left[\overset{\infty }{\underset{k=0}{\sum }}\gamma^{k}r_{t+k}|s_{t}=s,a_{t}=a\right]$$ (2)

The $Q^{*}\left(s,a\right)$ is corresponding to the optimal policy $\pi^{*}$, which can be represented as:


$$Q^{*}\left(s,a\right)=\underset{\pi }{\max}Q^{\pi }\left(s,a\right)$$ (3)

A $Q^{*}\left(s,a\right)$ follows the Bellman optimality equation:


$$Q^{*}\left(s,a\right)=E\left[r+\gamma \underset{a'\in A}{\max}Q^{*}\left(s^{\prime },a^{\prime }\right)|s,a\right]$$ (4)

In Q-learning, the Q-values for each state-action pair are stored and updated in a tabular form, which is well-suited for handling discrete and finite state spaces. However, it is not applicable for tasks with large or continuous state spaces as it can lead to the curse of dimensionality. DQN addresses the challenges of high-dimensional state space by incorporating deep neural networks as function approximators. Furthermore, DQN improves sample efficiency and training stability through techniques such as experience replay and target networks. The network parameters are updated to minimize the loss function during the training process, which is defined as:


$$L\left(\theta \right)=E_{s,a,r,s^{\prime }}\left[{\left(r_{t}+\gamma \underset{a_{t+1}}{\max}\hat{Q}(s_{t+1},a_{t+1}|\theta^{-})-Q(s,a|\theta )\right)}^{2}\right]$$ (5)

where *θ* is the parameter of the current network, and $\theta^{-}$ is for the target network. In DQN, the target of estimation $y_{t}$ is defined as:


yt=rt+γmaxat'Qtargetst+1,at';θ' (6)

where θ' is the parameter of $Q_{target}$. The detailed calculation process is shown in Appendix A in S1 File.

However, in DQN, the max operator applies the same values for selecting and evaluating an action, which elevates the likelihood of choosing overestimated values, consequently leading to excessively optimistic estimations of the values. To address this problem, Hasselt [33] introduced a strategy to disassociate the selection from the evaluation, and add a separate target network for calculating the target value $y_{t}$. The target of estimation $y_{t}$ in DDQN can be written as:


yt=rt+γQtargetst+1,argmaxat'Qestimationst+1,at';θ;θ' (7)

where $Q_{target}$ and $Q_{estimation}$ are the value of target network and estimation network, respectively.

Dueling DQN decomposes the Q-value function Q(s,a) into two separate estimators: one for the state value function $V\left(s\right)$ and another for the advantage function A(s,a). This decomposition allows the network to better differentiate between the intrinsic value of a state and the advantages of different actions within that state. In Dueling DQN, the Q-value is computed as follows:


$$Q\left(s,a;\theta \right)=V\left(s;\theta \right)+\left(A\left(s,a;\theta \right)-\frac{1}{\left|A\right|}\underset{a^{\prime }}{\sum }A\left(s,a^{\prime };\theta \right)\right)$$ (8)

where $\left|A\right|$ is the set of actions.

### DRL settings

#### Action space.

In this study, the agent selects its target toll lane by calculating expected rewards for each option, aiming to maximize the future global reward. In normal DRL-based lane-changing models, the action space typically consists of three actions (left, right, and keep current lane) for multi-lane scenarios, or two actions (change or keep current lane) in two-lane cases. However, at toll plaza diverging areas, the absence of lane markings results in no clear lane divisions. Therefore, the action space in this study is depended on the number of feasible toll lanes for the agents. The action space is defined by the toll collection type of the agent (either ETC or MTC) and the current arrangement of ETC and MTC lanes in the diverging area. Further constraints on the action space of AVs are detailed in the next section. The action space set *A* is described as follows:


$$A=\left\{\text{a|a }\in \text{feasible toll lanes}\right\}$$ (9)

In this study, we only considered the target toll lane selection of AVs with DRL, and the motions of AVs are controlled by the rules in the simulation platform. More detailed motion rules will be introduced in the next section.

#### State space.

**Fig 2:**
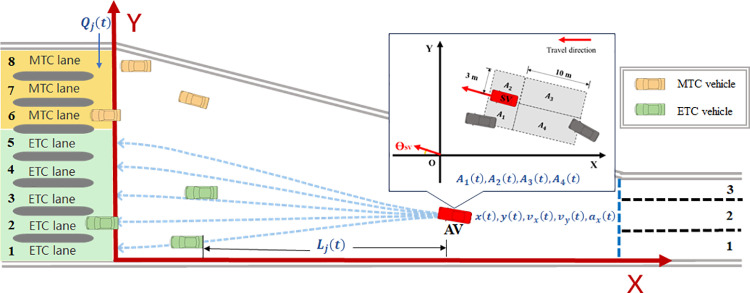
Illustration of surrounding environment.

According to the motivations and impacts of vehicles selecting and changing their target toll lanes at the toll plaza diverging area, the state space contains the state of the subject vehicle (SV) and the traffic condition:

(1) State of the SV: its position ($x\left(t\right)$ and $y\left(t\right)$), speed ($v_{x}\left(t\right)$ and $v_{y}\left(t\right)$), and acceleration ($a_{x}\left(t\right)$) at time step *t*;
(2) Surrounding environment: Due to the absence of lane markings at the toll plaza diverging area, previous lane-based environmental division is not feasible. To depict the surroundings of the SV, we defined a rectangular-shaped influence area and divided the area into 4 parts, including left ($A_{1}\left(t\right)$), right front ($A_{2}\left(t\right)$), left behind ($A_{4}\left(t\right)$), and right behind ($A_{3}\left(t\right)$), as show in Fig 2. The long side of the rectangle is always parallel to SV’s travel direction. Variables $A_{1}\left(t\right)$ to $A_{4}\left(t\right)$ are all binary variables, 1 for there is a vehicle in that area and 0 for there is none.
(3) Specific environment: The specific environmental information includes the number of vehicles queued in toll lane *j* ($Q_{j}\left(t\right)$), and the longitudinal distance SV can move after selecting toll lane *j* ($L_{j}\left(t\right)$) at time step *t*. *j* represents the numbering of the matching toll lanes for SV. For example, if the SV is an ETC vehicle and the toll plaza has three ETC lanes, then j=0,1,2. Variable $\beta_{j}\left(t\right)$ is defined as the ratio of the lateral distance from the SV’s current position to toll lane *j* to the longitudinal distance at time step *t*. A higher value of $\beta_{j}\left(t\right)$ indicates more aggressive lateral motion of SV for selecting toll lane *j*. Variable $L_{j}\left(t\right)$ represents the longitudinal distance from SV to the nearest vehicle on toll lane *j*. The method for calculating $L_{j}\left(t\right)$ and $\beta_{j}\left(t\right)$ will be explained in detail in the next section.

In conclusion, the state space of agent *i* at time step *t* is shown as:


$$s\left(t\right)=\left\{x\left(t\right),y\left(t\right),v_{x}\left(t\right),v_{y}\left(t\right),a_{x}\left(t\right),A_{1}\left(t\right),A_{2}\left(t\right),A_{3}\left(t\right),A_{4}\left(t\right),Q_{j}\left(t\right),L_{j}\left(t\right),\beta_{j}\left(t\right)\right\}$$ (10)

#### Reward function.

In most DRL-based decision-making models, the reward is determined by the vehicle’s state at the end of the lateral maneuver, without considering the execution process. However, it is incomplete to assess the effectiveness of a lateral motion solely based on a specific state. In this study, at first, the reward function is designed to reflect the driving efficiency during the entire diverging process. Second, decisions leading to collisions with surrounding vehicles will be penalized. Third, unnecessary lateral motion decisions, which increase driving safety risks, are also penalized. For instance, if the agent changes its target toll lane when there are no other vehicles ahead on this lane, a penalty should be applied. Based on the above considerations, the reward function in this study is set as follows:


$$R=R_{1}+R_{2}+R_{3}+R_{4}$$ (11)

where *ω* is the weighting factor. Given that vehicles need to decelerate in the diverging area to pass through the toll lanes, the reward function $R_{1}$ for efficiency is set as the negative value of the longitudinal distance from the AV to the toll lanes:


$$R_{1}=-\alpha_{1}\cdot L\left(t\right)$$ (12)

where $L\left(t\right)$ is the longitudinal distance from the agent to the toll lanes at time step*t*.$\alpha_{1}$is a weighting factor.

$R_{2}$ is used to encourage the agent to select a toll lane with shorter queue length. If the queue length of the new target is longer than that of the previous target, the AV will receive a penalty. Conversely, it will receive a reward.


$$R_{2}=\alpha_{2}(Q_{pre}\left(t\right)-Q_{new}\left(t\right))$$ (13)

where $Q_{pre}\left(t\right)$ and $Q_{new}\left(t\right)$ is the queue length of agent’s previous and new target toll lanes at time step *t*, respectively. $\alpha_{2}$ is a weighting factor.

$R_{3}$ penalizes the SV for collisions occurring during the lateral motion process:


$$R_{3}= \{\begin{array}{c} m_{3},Collision\\ 0,Otherwise \end{array}$$ (14)

$R_{4}$ penalizes the aggressive lateral motion:


$$R_{4}=-\alpha_{3}\cdot \beta$$ (15)

where *β* is the ratio of lateral distance from the agent’s current position to its new target toll lane to the value of $L\left(t\right)$. $\alpha_{3}$ is a weighting factor. Lateral motions often introduce disturbances into traffic flow. Especially in non-lane-based areas, the motions may have broader effects on surrounding vehicles. Thus, we designed $R_{4}$ to penalize aggressive target changes, with more rapid lateral motion resulting in greater penalties.

## Simulation platform

As previously mentioned, human driving behaviors at toll plaza diverging areas follow a destination-driven pattern with weak lateral constraints. The migration of car-following and lane-changing models designed for normal road segments cannot provide realistic interactions with HVs for the training of AVs. Therefore, it is crucial to develop a microscopic simulation model that can accurately reproduces the real human driving behaviors in this area. In this section, we proposed a two-dimensional micro-simulation method for HVs and integrated it into a simulation platform. A detailed description of the microscopic simulation model designed for HVs at toll plaza diverging areas is provided first, followed by an introduction to the training environment for AVs based on this platform.

### Microscopic simulation method

The “Perception-Decision-Action” (PDA) framework has been proven to be an effective approach for addressing complex interactions in traffic flow simulation [5]. This paper customizes the PDA framework specifically for the diverging areas, incorporating path-based perception, dynamic decision-making for target toll lane selection, and action rules for weak-constraint driving behaviors.

#### Perception layer.

In our simulation platform, vehicles are decentralized and self-driven. Each vehicle makes target lane selection decisions independently based on its own observation of the environment. AVs are not equipped with Vehicle-to-Everything (V2X) communication and rely solely on their own sensors for information in this paper. The information perceived by both HVs and AVs is the same, as described in state function in DRL settings, including self-related variables ($x\left(t\right),y\left(t\right),v_{x}\left(t\right),v_{y}\left(t\right),a_{x}\left(t\right)$), surrounding environmental variables ($A_{1}\left(t\right),A_{2}\left(t\right),A_{3}\left(t\right),A_{4}\left(t\right)$), and path-based variables ($Q_{j}\left(t\right),L_{j}\left(t\right),\beta_{j}\left(t\right)$). As the initial speeds of vehicles on the simulation platform are randomly set within a certain range, the environment around each vehicle is dynamically changing. In addition, the payment method on the highways depends on the driver’s own choice. Even vehicles with autonomous driving capabilities can choose the MTC. Therefore, the AVs in this study can be either MTC vehicles or ETC vehicles.

At the toll plaza diverging area, without the constraints of lane markings, vehicles often moving directly from their current position to the end of the queue at their target toll lane. Instead of perceiving the surrounding environmental information divided by lane markings, vehicles obtain the information about the accessible paths to their feasible toll lanes and use this information for subsequent target selections. Therefore, this paper employs two-dimensional smooth curves to simulate vehicle diverging paths and proposes a path-based perception method accordingly. Polynomial curves are commonly used to simulate lane-changing trajectories, as they generate trajectories with continuous curvature, ensuring steady changes in speed and acceleration. Cubic polynomials are used to simulate the diverging trajectories, expressed as:


$$f\left(x\right)=ax^{3}+bx^{2}+cx+d$$ (16)

where a,b,c represent parameters of the curve function respectively.

**Fig 3:**
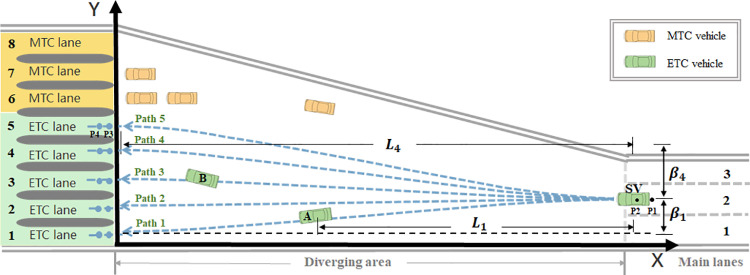
Illustration of path-based perception.

Specifically, each path is determined by four points, including the vehicle’s current and previous-step positions ($P_{1},P_{2}$) and two points on the median line of the toll lanes ($P_{3},P_{4}$), as shown in Fig 3. The variable $L_{j}\left(t\right)$ denotes the longitudinal distance the vehicle can move on path *j*. When other vehicles are on path *j*, $L_{j}\left(t\right)$ is the longitudinal distance between the SV and the nearest vehicle on path *j*. If not, $L_{j}\left(t\right)$ refers to the longitudinal distance from the vehicle to the toll lane. For example, $L_{1}$ is the longitudinal distance between SV and vehicle A, meaning the longitudinal distance SV can move for choosing path 1. Since no vehicles exist on path 4, $L_{4}$ is the longitudinal distance between SV and the toll lane 4. Vehicles within 0.8 m on either side of a path line are considered present on that path.

#### Decision layer.

The decision-making of AVs is based on the outcomes of DRL-based methods. For HVs, neural network (NN) is used to model the toll lane selection of HVs during the diverging process. NN is an effective method for classification or regression tasks as it can capture the complex relationships from data. In this study, a Multilayer Perceptron neural network is employed, and the parameters are optimized using the backpropagation algorithm.

#### Action layer.

**Fig 4:**
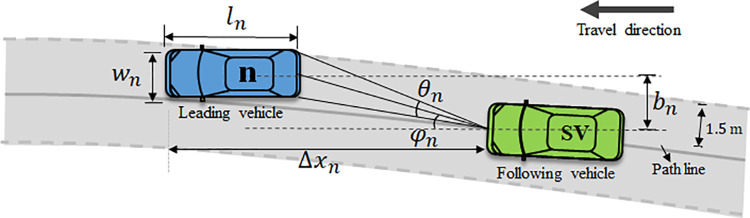
The car-following in weak lane-discipline traffic condition.

In the action layer, vehicles execute car-following rules on the paths selected in the decision layer. Unlike on normal road sections, the destination-driven driving characteristic lead to the centers of the leading and following vehicles existing lateral offsets. To address this issue, this study uses an improved Full Velocity Difference (FVD) model to describe car-following behavior in weak lane-discipline traffic conditions [34,35]. The vehicle with the shortest longitudinal distance to the subject vehicle (SV) and located within 1.5 m on either side of the SV’s current path centerline is considered as the leading vehicle, as shown in Fig 4.

The equation of the improved FVD is given as:


$$a_{SV}\left(t\right)=\alpha \left\{V\left[\Delta x_{SV}\left(t\right)\right]-V_{SV}\left(t\right)\right\}-\lambda_{1}\frac{d\theta \left(t\right)}{dt}+\lambda_{2}\frac{d\varphi \left(t\right)}{dt}$$ (17)

where $V\left[\Delta x_{SV}\left(t\right)\right]$ denotes the driver’s optimal velocity prefers based on the distance headway; *α* represents the sensitivity of the driver to the difference between the optimized velocity and its own velocity; $V_{SV}\left(t\right)$ is the speed of SV; $\lambda_{1}$ and $\lambda_{2}$ are the sensitivity coefficients. $\theta \left(t\right)$ is the visual angle, and $\varphi \left(t\right)$ is the offset angle, calculated as follows:


$$\theta \left(t\right)=arctan\frac{b_{n}\left(t\right)+w_{n}/2}{\Delta x_{n}\left(t\right)-l_{n}}-arctan\frac{b_{n}\left(t\right)-w_{n}/2}{\Delta x_{n}\left(t\right)-l_{n}}$$ (18)

$$\varphi \left(t\right)=arctan\frac{b_{n}\left(t\right)}{\Delta x_{n}\left(t\right)-l_{n}}$$ (19)

where $l_{n}$ and $w_{n}$ are the length and width of the leading vehicle, respectivelsy. In this paper, the length and width of all vehicles are set to 5m and 1.6m.

And the corresponding optimized velocity function $V\left[\Delta x_{SV}\left(t\right)\right]$ is given as:


$$V\left[\Delta x_{SV}\left(t\right)\right]=V_{1}+V_{2}\text{tanh}\left(C_{1}\frac{b_{n}\left(t\right)}{\tan\varphi_{n}\left(t\right)}+C_{2}\right)$$ (20)

where $V_{1}$, $V_{2}$, $C_{1}$, and $C_{2}$ are parameters need to be calibrated. The values of the four parameters are set as $V_{1}=6.75$, $V_{2}=7.91$, $C_{1}=0.13$, $C_{2}=1.57$ in this study. Furthermore, a virtual vehicle will be positioned at SV’s target toll lane to facilitate the its car-following behavior when there are no leading vehicles ahead of it.

#### Study site.

**Fig 5:**
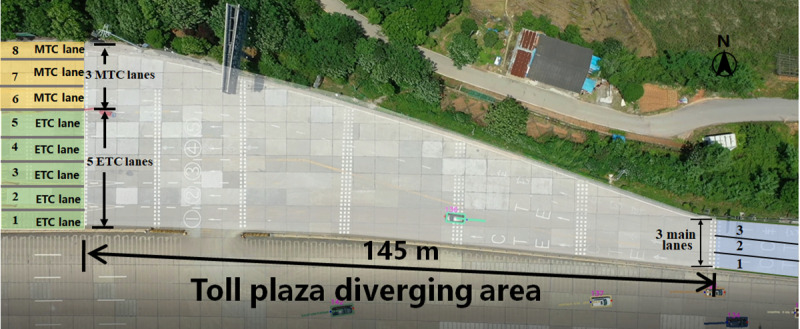
An aerial view of the toll plaza diverging area (captured by UAV).

The data used for simulation model calibration was obtained from the real-world vehicle trajectories collected at a toll plaza diverging area on the G55 freeway in Changsha, China. This east-west freeway has three lanes in each direction and serves as a major corridor in western Changsha city. As shown in Fig 5, the diverging area are 145 m long and has 5 ETC lanes on the left side and 3 MTC lanes on the right side. The trajectories were recorded using an Unmanned Aerial Vehicle (UAV) with 4K ultra-high-definition video at 30 frames per second (fps). Detailed calibration and simulation accuracy of the microscopic simulation method are presented in another manuscript currently under review. Since this paper focuses on the DRL-based strategies for AVs, further calibration and simulated accuracy are included in the Appendix B.

#### Training environment.

**Fig 6:**
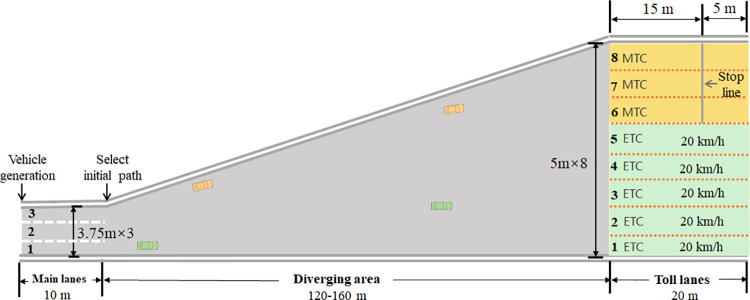
The visual interface of the simulation platform.

The platform integrating the proposed microscopic simulation method is implemented in Python language and consists of three subsystems: visual interface, simulation computation, and data logging. The visual interface of the simulation platform is shown in Fig 6. The traffic flow is 1,500 vehicles per hour, including ETC vehicles and MTC vehicles. Vehicles are generated on the main lane segment, 10 m before the diverging area. The main lane segment has three lanes, each 3.75 m wide. Both the ETC and MTC toll lanes are 5 m wide.

The initial speeds of ETC and MTC vehicles follow normal distributions of (14.7, 3) m/s and (12, 3) m/s, respectively. The departure ratio of ETC vehicles from main lanes 1, 2, and 3 is 1:2:1, while the departure ratio for MTC vehicles is 1:2:4. The number of ETC vehicles is approximately twice that of MTC vehicles. ETC vehicles need to pass through the toll lanes at 20 km/h, while MTC vehicles must stop 15–20 seconds at 15 m inside the MTC toll lanes to simulate manual toll collection. It should be noted that all the above scenario settings are consistent with the traffic information in the collected trajectory data. When applied to other diverging areas, the parameters of the microscopic simulation model must be recalibrated based on local trajectory data and geometric features.

As drivers have the option to choose their payment methods, both AVs and HVs on our platform can be either ETC or MTC vehicles. All vehicles simulated in this platform are cars. The duration of AV’s every toll selection decision is 1 s. The action layer updates the vehicle states once per 0.1 s to control the vehicle movements. In each episode, only one AV enters the diverging area. When the AV reached the toll lane or collided, the episode ended, and the next episode began. Once the AV completes a diverging process, we randomly picked one HV at the origin point as a new AV and the target selection strategy is inherited by the new AV for further improvement. The duration of each episode approximately falls between 8 and 15 seconds.

Durning the training process, the agent employs a random exploration strategy, *ε* -greedy, to balance exploration and exploitation, with a threshold of 0.9. For the hyperparameter settings of the DRL methods, the learning rate is set to 0.001, the memory size for experience replay is 20,000 samples, the batch size is 128, and the discount factor *γ* is 0.98. The number of neurons in both the first and second hidden layers is 64. The target Q-network is updated every 500 steps. For each DRL model, ETC AVs were trained over 20,000 episodes, and MTC AVs over 15,000 episodes. The search ranges for hyperparameters and the sensitivity analysis can be found in Appendix C. The entire procedure was conducted on a computer with a 2.30 GHz i7-12700H CPU, 32.0 GB of RAM and NVIDIA GeForce RTX 3070 Ti GPU. The program is implemented based on Python 3.8 and the neural network used Tensorflow 2.6.0.

## Results and discussion

### Lateral motion strategy for a single AV

**Fig 7:**
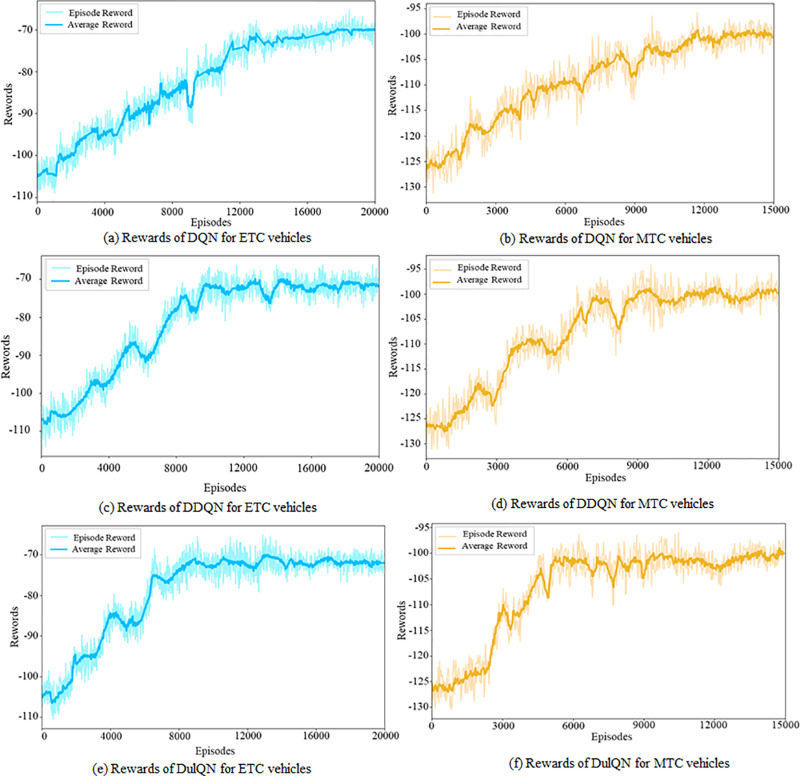
Average reward variation of three models.

Considering that MTC and ETC vehicles have different target toll lanes, the lateral motion decisions for AVs with the two toll collection types are separately trained using the DQN, DDQN, and Dueling DQN models. Fig 7 depicts the overall rewards of the three models, with the left side displaying training results for ETC vehicles and the right side for MTC vehicles. Multiple trials were conducted to ensure model robustness, with average rolling rewards represented by solid lines and fluctuating amplitudes depicted with shaded areas. The learning speeds of the DDQN and Dueling DQN models are faster than that of the DQN model, and the Dueling DQN model shows a better training stability. Since MTC vehicles must travel a greater distance in the diverging area, their overall reward value is lower than that of ETC vehicles.

The background traffic flow settings in the simulation platform are same with those in Section Training environment. Considering the randomness in the generation of background traffic flow, the average diverging speed of individual trained AVs over a one-hour simulation is chosen as the evaluation indicator, as it minimizes the influence of individual outliers and reflects the overall effectiveness of the methods. In each simulation, a single AV is introduced sequentially. Once the AV completes this process, the next AV is generated at the starting point. And the same number of ETC HVs and MTC HVs are collected as control groups. 500 vehicles were collected for each group: ETC AVs (DDQN), ETC AVs (Dueling DQN), MTC AVs (DDQN), MTC AVs (Dueling DQN), ETC HVs, MTC HVs. Furthermore, to validate the effectiveness of the proposed strategy under varying traffic scenarios, four traffic flow scenarios were set with hourly flows of 1250, 1500, 1750, and 2000 vehicles. 1500 vehicles per hour is the actual traffic flow at our study site.

**Fig 8:**
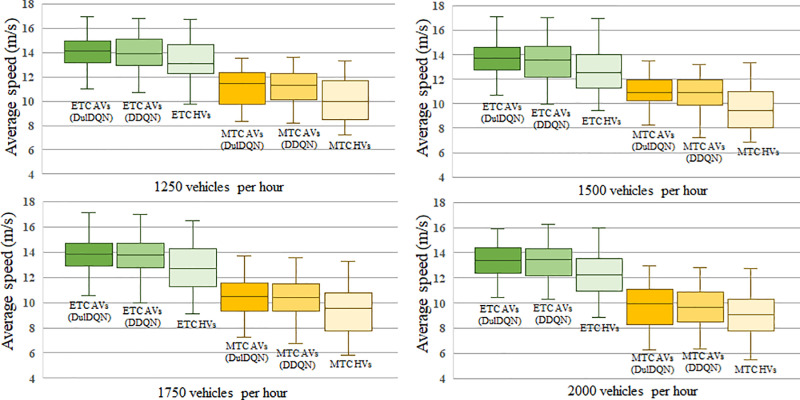
The average diverging speeds of AVs under different traffic flows.

The comparison of average diverging speeds between these groups is shown in Fig 8. The results show that as traffic flow increases, the average diverging speed of both AVs and HVs decreases. However, under all traffic scenarios, the average travel speeds of trained AVs are higher than those of HVs for both ETC and MTC vehicles, with a more concentrated speed distribution and an elevated lower bound. This demonstrates that under varying traffic conditions, the proposed lateral motion strategy improves travel efficiency in diverging areas, highlighting its robustness. Moreover, AVs trained with the DuelDQN model achieved the overall highest average travel speed with relatively lower variance, indicating more stable training outcomes.

### Impacts of penetration rate on traffic flow

Next, we investigate the impact of an increasing AV penetration rate using this self-efficient strategy on traffic flow in the diverging area. According to the results of single AV, the strategy based on DuelDQN is utilized for AVs due to its superior performance in enhancing travel efficiency. As the AV penetration rate increases from 0% to 100%, each simulation runs for 1 hour with the same simulation settings as in Section Training environment. The average travel time and the traffic conflict indicator Post Encroachment Time (PET) are employed for evaluating driving efficiency and safety. Note that AVs here include both ETC and MTC AVs.

**Fig 9:**
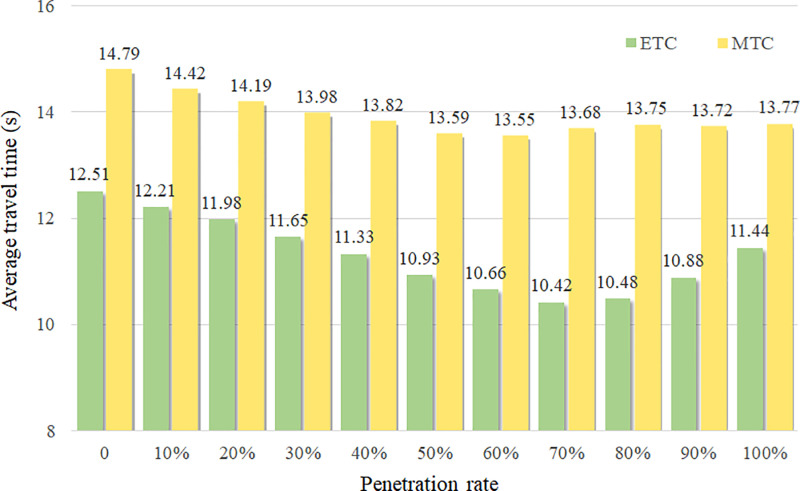
The average travel time of vehicles under different penetration rates.

Fig 9 illustrates the average travel time distribution for ETC and MTC vehicles in the diverging area under varying AV penetration rates. For ETC vehicles, when the penetration rate is less than 70%, their average travel time decreases, indicating that the introduction of AVs has increased the travel efficiency. Within the [70%, 80%] range, the average travel time remains stable, followed by an increase beyond 80% penetration. MTC vehicles display a similar trend, with a turning point between [50%, 60%]. When the penetration rate is larger than 60%, the average travel time of MTC vehicles shows a slight increase and then stabilizes. The earlier inflection point in the average travel time for MTC vehicles may because their fewer accessible toll lanes than ETC vehicles. This limited number of accessible toll lanes restricts AVs’ ability to improve traffic efficiency.

**Fig 10:**
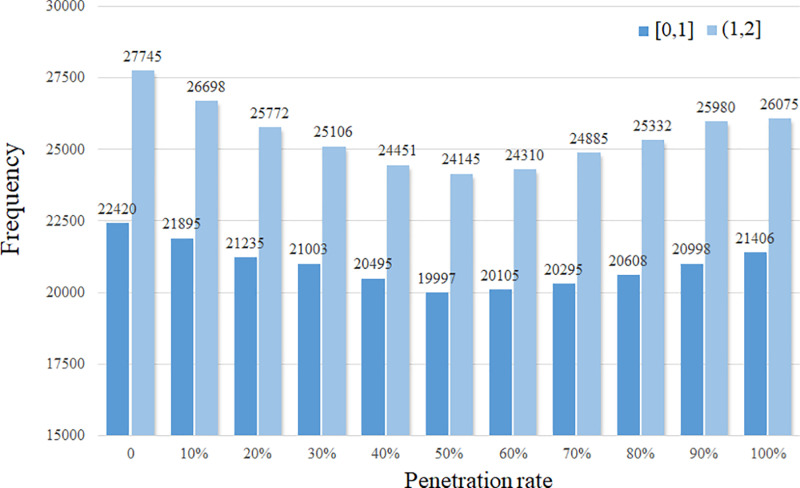
The traffic conflicts under different penetration rates.

Fig 10 depicts the changes in vehicle conflicts as the AV penetration rate increases. Calculating conflict risk is a commonly used proactive safety assessment method. Compared to historical crash data, it is more observable and better suited for identifying potential risks [36]. As previously discussed, in the non-lane-based traffic areas, vehicles may have conflicts with others at any angel. However, traffic conflict indicators such as Time-to-Collision (TTC) and Deceleration Rate to Avoid a Crash (DRAC) are primarily designed to evaluate conflicts between consecutive vehicles in the same traffic lane, which are unsuitable for assessing crossing conflict events. Therefore, the indicator PET is employed in this study for safety assessment. PET is defined as the time difference between the departure of the first vehicle from the conflict point and the arrival of the second vehicle at the same point. The threshold is set as 2 seconds in this study. For a more detailed analysis, PET values are categorized into two intervals: [0,1] and (1,2] seconds, representing different conflict levels. The figure indicates that as the penetration rate increases from 0% to 50%, the number of conflicts in both [0,1] and (1,2] intervals decrease. HVs generally prefer toll lanes with shorter required lateral distance, while the AVs’ lateral motion strategy leads to a more balanced selection of target toll lanes. However, when penetration exceeds 50%, conflicts within the (1,2] interval increase obviously, and beyond 80% penetration, the number of severe conflicts within the [0,1] interval show a marked rise. As shown in Figs 9 and 10, a higher penetration of AVs based on self-efficient strategy not always improve the traffic efficiency at toll plazas. A high proportion of AVs with a self-efficient strategy may lead to intensified competition for limited road resources and the deterioration of traffic safety conditions.

## Conclusion

Existing research on AV decision-making has mainly focused on normal road sections, with limited exploration of decision-making capability in complex traffic areas without lane markings. This study selects a typical non-lane-based complex traffic area, the toll plaza diverging area, to develop an efficient lateral motion strategy for AVs. First, we developed a microscopic simulation platform that can accurately reflect HVs’ diverging trajectories, providing realistic interactions for AVs. Second, we designed a reward function tailored to the environmental features of the diverging area and proposed a DRL-based self-efficient lateral motion strategy. The results demonstrate that the AV with the proposed self-efficient lateral motion strategy has higher travel efficiency than HVs in the diverging area.

Currently, the intelligent driving or advanced driver-assistance systems of vehicles make lateral motion decisions to maximize their individual benefits. It is essential to consider their potential impacts on traffic flow, particularly in complex traffic areas. Thus, we further explored how the varying penetration of AVs with self-efficient strategy impacts traffic flow in the diverging area. Findings show that certain penetration rate of AVs with this strategy indeed improves the overall traffic efficiency and safety of the diverging area. However, when the number of AVs adopting this strategy exceeds a threshold, both efficiency and safety tend to decline due to their competition for limited road resources.

Therefore, there are two directions that require further exploration in the future. First, the proposed strategy is designed for improving single-vehicle efficiency and does not account for collaboration among multiple AVs. In scenarios where multiple AVs coexist, a cooperative strategy may further enhance both safety and efficiency by enabling vehicles to share information and coordinate their motions. Second, this study primarily focuses on the lateral motion decisions of AVs, without optimizing their longitudinal motions. Longitudinal motion control, such as speed and acceleration optimization, is also critical for ensuring efficient and comfortable motions in complex traffic areas. Future research will aim to integrate the optimization of both lateral and longitudinal motions to provide a more comprehensive decision-making framework for AVs.

## Supporting information

S1 File Appendix. (DOCX)
