# Supplementary material for: Deep reinforcement learning for decision making of autonomous vehicle in non-lane-based traffic environments
Source: PLoS One. 2025 Apr 16;20(4):e0320578. doi: 10.1371/journal.pone.0320578 (PMC12002507; doi:10.1371/journal.pone.0320578)
Supplement: S1 File — (DOCX) [file pone.0320578.s001.docx]

**Appendix A**

| **Algorithm:** Pseudo-Code of the DQN-based methods for decision making of AVs |
| --- |
| Initialize experience replay buffer D;  Initialize $Q$ network with random weights $Q$ and target network $Q^{-}$ with weights $\theta^{-}=\theta$;  **for** $episode=1,2\ldots N$ **do**  Reset environment and initial state $\phi(s_{0})$ from the simulation platform;  **for** $t=1,2\ldots T$ **do**  Use $\phi(s_{t})$ as input of $Q$ network and get $Q$ value of each action;  Choose action $a_{t}=\epsilon-greedy(s_{t})$;  Take action $a_{t}$ under $s_{t}$, observe reward $R$ and new state $\phi(s_{t+1})$;  Push $(\phi\left( s_{t} \right), a_{t}, R, \phi(s_{t+1})$) into D;  Randomly take m samples $(\phi\left( s_{j} \right), a_{j}, R, \phi(s_{j+1})$) from D;  Compute approximate value:$\phi\left( s_{j+1} \right) is terminal$ $\phi\left( s_{j+1} \right) is nonterminal$  $y_{j}=\left\{ \begin{aligned} R_{j} \\ y_{nt} \end{aligned} \right.\begin{matrix} \phi\left( s_{j+1} \right) is terminal \\ \phi\left( s_{j+1} \right) is nonterminal \end{matrix}$  where $y_{nt}=R_{j}+\gamma\max_{\boldsymbol{a}} Q^{-}(\phi\left( s_{j} \right),a;\theta^{-})$  Update $Q$ network by executing gradient decent on loss function:  $L\left( \theta\right)=1/m\sum_{j=1}^{m} {(y_{j}-Q(\phi\left( s_{j} \right), a_{j};\theta))}^{2}$  Set $\theta^{-}= \theta$ every C steps;  **end**  **end** |

**Appendix B**

The effectiveness of the “Perception-Decision-Action” simulation method used in the simulation platform is presented in the appendix. The appendix includes the prediction accuracy of the decision layer for the target toll lane selection, as well as the trajectory simulation accuracy of the action layer.

*The accuracy of the decision layer*

According to our study site introduced above, the trajectories of 692 vehicles were extracted, consisting of 628 cars, and 64 buses and trucks. The traffic volume in the diverging area was approximately 1,500 vehicles per hour (calculated as 6 times of the 10-min volume interval). Given the low proportion of buses and trucks, this study focuses solely on cars, including 439 ETC cars and 189 MTC cars. Hereafter, "vehicles" refers to cars.

Upon entering the diverging area, each vehicle selects an initial target toll lane and then considers whether to change its target during the diverging process. The Wavelet Transform is applied to detect the target changes from vehicle trajectory data, with further details available in in our previous study [1]. The model assumes vehicles reconsider target selection when encountering other vehicles ahead on their current path. Based on this assumption, a total of 18,232 observations of target lane selection were recorded, including 628 initial target selection samples of each vehicle and 17,604 target selection samples during the diverging process. The current target toll lane of the SV is considered as the path selection result at that moment. The table B1 presents the classification accuracy of the NN model on both training and test sets. The model achieved an accuracy over 90% in predicting toll lane selection for both ETC and MTC vehicles.

Table B1 The accuracy of NN model

| Type | ETC | MTC |
| --- | --- | --- |
| Training set | 0.9889 | 0.9893 |
| Test set | 0.9886 | 0.9875 |

*The accuracy of the action layer*

In this study, parameters of the improved FVD car-following model were calibrated using the Particle Swarm Optimization (PSO) algorithm [2, 3], with a time step of 0.1 s. Following Tang [4], both the self-learning and social-learning coefficients of PSO were set to 2, and the inertia weight to 0.9. The particle swarm size and the number of iterations were set to 15 and 20, respectively. Additionally, we compared the calibration results of the Genetic Algorithm (GA) and Hybrid Evolutionary Algorithm (HEA) with PSO. When no leading vehicle is present for the subject vehicle (SV), virtual vehicles were positioned at the toll booths to ensure the SV could complete the car-following behavior. The indicators root mean square error (RMSE) and Mean Absolute Error (MAE) are adopted to quantify the deviation between the simulated and actual trajectories. The average RMSE and MAE of ETC and MTC vehicles based on different methods are shown in the Table B2.

Table B2 Estimation accuracy of three models

| Method | ETC vehicles | | MTC vehicles | |
| --- | --- | --- | --- | --- |
|  | Average MAE/(m) | Average RMSE/(m) | Average MAE/(m) | Average RMSE/(m) |
| GA | 0.4576 | 0.6752 | 0.3302 | 0.5829 |
| PSO | 0.3386 | 0.5883 | 0.2515 | 0.4779 |
| HEA (PSO+TS) | 0.3403 | 0.5705 | 0.2489 | 0.4723 |

**Appendix C**

Taking the training of ETC AVs with the DQN algorithm as an example, Table C1 presents the average reward per episode after stabilization for each tested hyperparameter value. This table reflects the impact of different hyperparameter settings on model performance.

Table C Sensitivity Analysis Results for Hyperparameter Tuning

| **Hyperparameter** | **Range** | **Average Reward for one episode** |
| --- | --- | --- |
| Learning rate | 0.01, **0.001** | -71.16，-70.15 |
| Replay Memory Size | 10000, **20000**,30000 | -73.05，-70.14，-69.95 |
| Batch Size | 64, **128**, 256 | -70.34，-70.42，-70.16 |
| Discount Factor | 0.95, **0.98**, 0.99 | -71.06，-70.53，-70.67 |

**References**

1. Long K, Fei Y, Xing L, Pei X, Yao D, Zheng O, et al. Predicting vehicle trajectory of non-lane based driving behaviour with Temporal Fusion Transformer. Transportmetrica B: Transport Dynamics. 2024;12(1):2326018. doi: 10.1080/21680566.2024.2326018.

2. Akopov AS, Beklaryan LA, Beklaryan AL. Simulation-Based Optimisation for Autonomous Transportation Systems Using a Parallel Real-Coded Genetic Algorithm with Scalable Nonuniform Mutation. Cybernetics and Information Technologies. 2021;21(3):127–44. doi: 10.2478/cait-2021-0034.

3. Haris M, Nam H. Path Planning Optimization of Smart Vehicle With Fast Converging Distance-Dependent PSO Algorithm. IEEE Open J Intell Transp Syst. 2024;5:726-39. doi: 10.1109/OJITS.2024.3486155.

4. Tang L, Zhang D, Han Y, Fu A, Zhang H, Tian Y, et al. Parallel-Computing-Based Calibration for Microscopic Traffic Simulation Model. Transp Res Rec. 2023;2678(4):279-94. doi: 10.1177/03611981231184244.
